# Recalcitrant Fungal Corneal Ulcer

**DOI:** 10.7759/cureus.30866

**Published:** 2022-10-30

**Authors:** Dharshini Gounder, Archana Thool

**Affiliations:** 1 Ophthalmology, Jawaharlal Nehru Medical College, Datta Meghe Institute of Medical Sciences, Wardha, IND

**Keywords:** slit lamp, keratitis, therapeutic keratoplasty, perforation, fungal corneal ulcer

## Abstract

The cornea forms the major refracting surface of the eye. Any disease affecting the cornea leads to severe visual impairment. One of the most common pathologies affecting the cornea is infective keratitis. In India, especially in rural areas, infective corneal ulcers are major causes of visual impairment. Medical therapy with antimicrobial medicines is usually started first and if fails to heal the ulcer or there is progressive corneal thinning or corneal perforation then surgical treatments like penetrating keratoplasty are the only modality to salvage the globe integrity. Fungal keratitis particularly in tropical and subtropical areas has a poor prognosis as compared to bacterial keratitis. The main reasons for poor outcomes are delayed diagnosis and a lack of effective antifungal therapies. Over the last decade, significant advances have been made in quick diagnosis in cases of mycotic keratitis and also effective medical treatment. We report a case of a 56-year-old female who was diagnosed with left eye fungal corneal ulcer on systemic and topical antifungal agents for the last two months. She presented to us with an increase in pain, watering, and redness in her left eye for the past two days. On examination, the patient had desmetocele with impending perforation. The patient was advised to undergo therapeutic penetrating keratoplasty in her left eye. Penetrating keratoplasty plays a crucial part in eyes with refractory keratitis, impending perforation, or eyes with corneal perforation. Penetrating keratoplasty in such cases helps to replace the diseased tissue, decrease the infective load, and also helps to restore anatomical integrity of the globe and usable eyesight with good success rate. Early intervention prior to perforation or limbal/scleral extension can lead to better results at least in maintaining the globe integrity.

## Introduction

Infective keratitis is the second most common aetiology of blindness after cataracts in developing countries. A corneal ulcer is a loss of corneal tissue typically accompanied by inflammation, and ulcerative keratitis is the umbrella name for a collection of diseases that contribute to ulceration [1]. According to the World Health Organization, corneal ulceration causes around 1.5 to 2 million new cases of monocular blindness each year in underdeveloped countries [2]. The primary pathology for ulceration includes a breach in epithelium followed by stromal involvement and its destruction by microbial agents. Because of its known and mysterious aetiology, corneal ulcer management remains a challenge for ophthalmologists [3]. Corneal ulcers frequently result in scarring, astigmatism, and substantial vision loss. Perforation, scleral involvement, and endophthalmitis may occur in severe cases [4]. However, prompt management can help to reduce the damage and enhance the outcome. Infectious ulcers typically heal with antimicrobial treatment, but ulcers that are on the verge of perforation require immediate surgical intervention like conjunctival flaps, tissue adhesives, or therapeutic penetrating keratoplasty [5]. Bacteria, viruses, fungi, and parasites can all cause corneal ulceration. Depending on the geographic area, fungal corneal ulcers account for 1-44 per cent of all instances of microbial keratitis [6]. The most predisposing factors for fungal corneal ulceration are ocular trauma by vegetative material, prolonged steroid usage, and compromised ocular surface [7]. Fungal corneal ulcers are commonly seen following trauma by vegetative material or in people working in the soil. They cause severe ocular morbidity and are challenging to treat compared to bacterial ulcers. This infection should be treated as soon as possible to reduce morbidity and further consequences like corneal perforation [8]. A severe and deep fungal corneal ulcer can lead to corneal perforation.

Various factors which can lead to perforation are excessive alcohol use, the central location of the ulcer, lack of corneal vascularization, delay in initially starting the treatment, failure to start fortified medications (antibiotics), exposure to keratitis or corneal dryness [9]. The substantial ocular morbidity of corneal perforations necessitates quick identification and treatment. Surgical intervention is required whenever a corneal ulcer is unresponsive to medical management or there is impending perforation. It may be in the form of bandage contact lenses, tarsorrhaphy, or therapeutic penetrating keratoplasty [10]. Penetrating keratoplasty is the commonly performed procedure to maintain anatomical integrity. Amniotic membrane transplantation has proven to be a viable supplementary approach for corneal re-epithelization; however, it has not yet replaced Keratoplasty, owing to donor tissue availability. Although penetrating keratoplasty is a well-known treatment, long-term complications like postoperative reinfection, astigmatism, retinal detachment, immunological reactions, and graft failure continue to be major factors hindering fruitful outcomes. Corneal ulceration was not previously thought to be a significant cause of corneal blindness because it usually affects one eye of the affected person. These individuals are not classified as completely blind but rather as visually impaired.

## Case presentation

**Figure 1:**
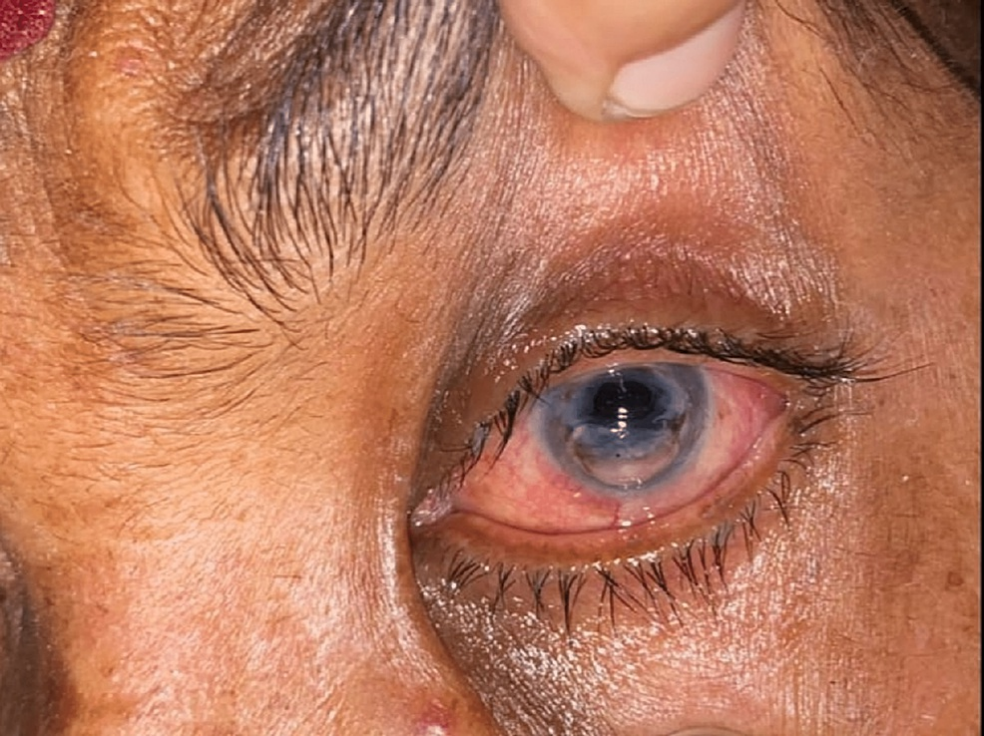
Preoperative image

We report a case of a 56-year-old woman to our outpatient department of ophthalmology with chief complaints of redness, pain, and watering in her left eye for the last two days. The patient was diagnosed with a case of a fungal corneal ulcer for two months and was on systemic tablet ketoconazole 200mg BD; topical suspension natamycin 5% one hourly, eye drop timolol (0.5 %) BD, eye drop moxifloxacin (0.5%) six times a day and eye ointment atropine (1%) BD. The patient had corneal scraping with KOH potassium hydroxide) mount report which was suggestive of filamentous fungi. The patient denied having had any ocular injuries but gave a history of constantly rubbing her left eye as she was experiencing foreign body sensations. Ophthalmic examination showed visual acuity of hand movement close to face, with projection of rays accurate in all four quadrants in the left eye. The slit lamp examination showed swollen upper and lower eyelids, circumcorneal congestion, diffuse thinning of cornea with infiltrates extending up to limbus (Figure 1). Also, there was desmetocele of the central and inferior cornea with clear lens abutting the cornea. Pupil was mid dilated non reacting to light. Sac syringing was done to reveal patent lacrimal passage on both sides. Right eye ocular examination was normal, with the best corrected visual acuity of 6/6.

**Figure 2:**
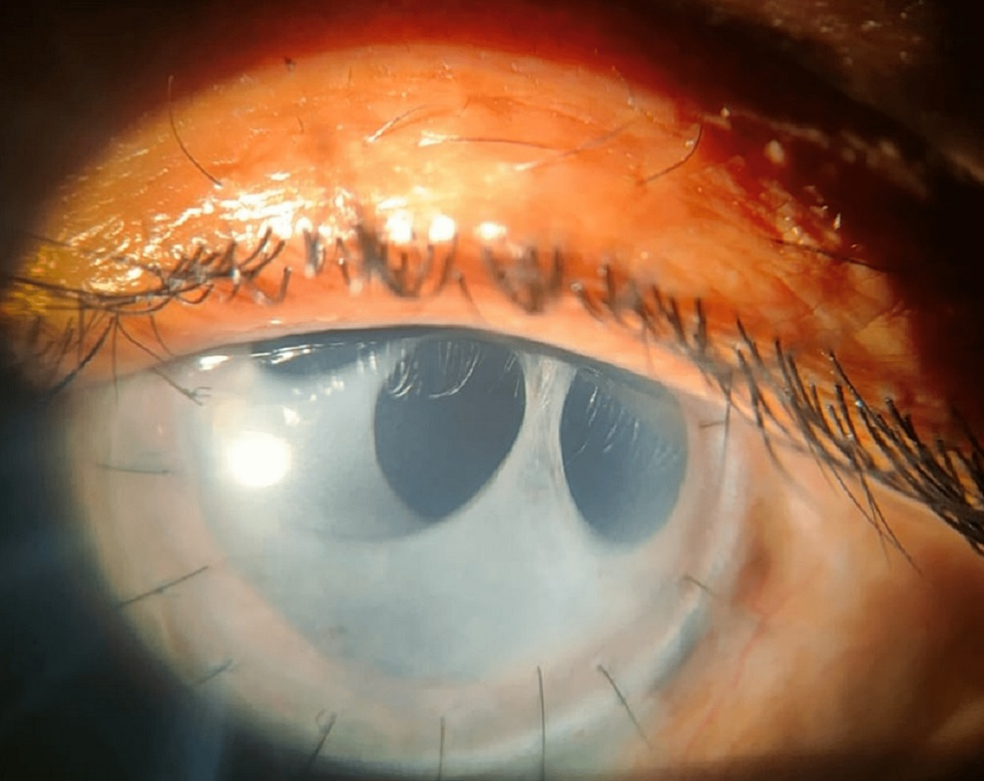
Postoperative image at three months follow-up

A normal complete blood count and erythrocyte sedimentation rate (ESR), a normal urine specimen, and regular immunological markers were discovered during the laboratory examination. In view of impending perforation, corneal scraping was avoided. The patient was started on ceftriaxone 1 g/day intravenously twice a day, pantoprazole intravenously, and diclofenac twice a day. Also, tablet acetazolamide 250mg twice a day was given. Topical medication included moxifloxacin four hourly/day, fortified vancomycin one hourly, fortified voriconazole one hourly, continued eye ointment atropine twice a day, and eye drop timolol (0.5%) twice a day. While the patient was waiting for therapeutic penetrating keratoplasty, on day three of admission, the patient developed corneal perforation. Meanwhile, a bandage contact lens was given to the patient, followed by therapeutic penetrating keratoplasty under the peribulbar block, which was performed on the left eye as an emergency procedure. Intraoperative before the start of the procedure, lens extrusion was noted. The corneal tissue excised was almost till limbus. The whole iris which was adherent to the cornea was removed, so total iridectomy was done; only the posterior capsule in the nasal and inferior quadrant was left behind. The eye was left aphakic after a 9.5 mm corneal transplant and was sutured with 10-0 nylon five with 16 interrupted sutures. The removed corneal button was sent for culture report but showed no growth; it may be that the patient was already on antifungal medication. The patient had a visual acuity of 1 m on the first postoperative day. All sutures were intact, the graft was clear, and the graft-host interface was properly opposed. The patient was started on topical suspension natamycin six times a day, eye drop moxifloxacin six times a day, eye drop atropine twice a day, and eye drop timolol twice a day. Systemic oral tablet cefixime 200 mg twice a day was continued for five days. After corneal button report showed no growth, she was started on oral tablet prednisolone 60 mg in tapering dose every seven days along with topical eye drop prednisolone acetate (1%) six times a day. The graft was clear after two weeks. Till three months postoperative graft tissue was clear with no signs of reinfection or graft rejection (Figure 2). The patient was planned for secondary intraocular lens (IOL) implantation at a later date.

## Discussion

Fungal keratitis is endemic in developing countries like India. It’s a major public health concern in our country due to limited access to health care and economic barriers. Early diagnosis and timely intervention are key to preventing visual loss in such cases due to opacity and complications like perforation. With the availability of various topical and systemic antifungals, it is possible to treat these cases effectively if diagnosed timely. The most recent available antifungal drugs have barriers, for instance, low bioavailability and ocular penetration, which are particularly problematic in situations with deep-seated infections [11]. As a result, penetrating keratoplasty is regarded as the most effective therapy for eyes with deep, non-healing ulcers, impending perforations or perforated corneal ulcers. Alternative approaches including tenon patch grafting, autologous fibrin membrane grafting with solid platelet-rich plasma, and grafting with processed pericardium mixed with synthetic material, as well as partial thickness lamellar grafting techniques, have lately sparked attention [12,13]. Penetrating keratoplasty plays a key role in the treatment of severe and refractory keratitis, with a high rate of success in restoring anatomical integrity and restoring usable vision. Our patient diagnosed with a case of a fungal ulcer was on medications. However, there was no improvement in her symptoms and she presented to us late in the course with impending perforation. Thus surgical intervention in the form of penetrating keratoplasty was the only viable option to maintain the globe's integrity. Intraoperative findings of adherent iris to cornea and lens extrusion with poor capsular support left but with the only chance of doing therapeutic keratoplasty without IOL implantation. Also, the graft size taken was large due to involvement up to limbus. Follow-up after three months revealed that the corneal graft was clear with intact sutures. There were no signs of recurrence of infection or graft rejection.

## Conclusions

Infective corneal ulcer is one of the major causes of corneal blindness. Because of the chronic course of the disease and poor compliance of the patient with medications, patients come with complications like perforation. Penetrating keratoplasty remains the gold standard in eyes with perforated corneal ulcers for anatomical restoration and useful vision. However early intervention before perforation or limbal or scleral expansion may result in an anatomically and visually improved outcome.
